# The fragility index may not be ideal for paediatric surgical conditions: the example of foetal endoscopic tracheal occlusion

**DOI:** 10.1007/s00383-021-04926-x

**Published:** 2021-05-29

**Authors:** Arne Schröder, Oliver J. Muensterer, Christina Oetzmann von Sochaczewski

**Affiliations:** 1 Klinik für Kinder-und Jugendmedizin, Klinikum Dortmund, Dortmund, Germany; 2 Klinik für Kinder-und Jugendmedizin, Elisabeth-Krankenhaus Essen, Essen, Germany; 3 Klinik und Poliklinik für Kinderchirurgie, Dr. von Haunersches Kinderspital, Ludwig-Maximilians-Universität München, München, Germany; 4 Klinik und Poliklinik für Kinderchirurgie, Universitätsmedizin der Johannes-Gutenberg-Universität Mainz, Mainz, Germany; 5 Sektion Kinderchirurgie, Klinik und Poliklinik für Allgemein, Viszeral, Thorax und Gefäßchirurgie, Universitätsklinikum der Rheinischen Friedrich-Wilhelms-Universität Bonn, Venusberg-Campus 1, D-53127 Bonn, Germany

Sir,

We truly appreciate that *Saikia* & *Thakuria* have found the time during this devastating pandemic to respond to our manuscript. It is a pleasure for us to respond to their comments to the editor in this issue of *Paediatric Surgery International* [1], which provides us with the valuable opportunity to point out some issues that may not have become clear enough in our initial contribution [2].

We feel that transparency is crucial within the scientific discourse [3–6], and we agree that the search strategy should be readily available. The list of included meta-analyses and the extracted data [7] have been put into a repository and cited in reference 35 of our initial contribution, so that the readership is able to double-check and reproduce our results.

The included meta-analyses have purposefully not been limited to studies with a 1:1 allocation ratio. The fragility index has been extended beyond this limitation before [8–10], and we have thus extended its applicability as well. Let us assume that we would have limited our analysis [2] to meta-analyses with these primary study properties: the underlying numbers would have possibly changed slightly, but it would not have changed our conclusion.

**Fig. 1:**
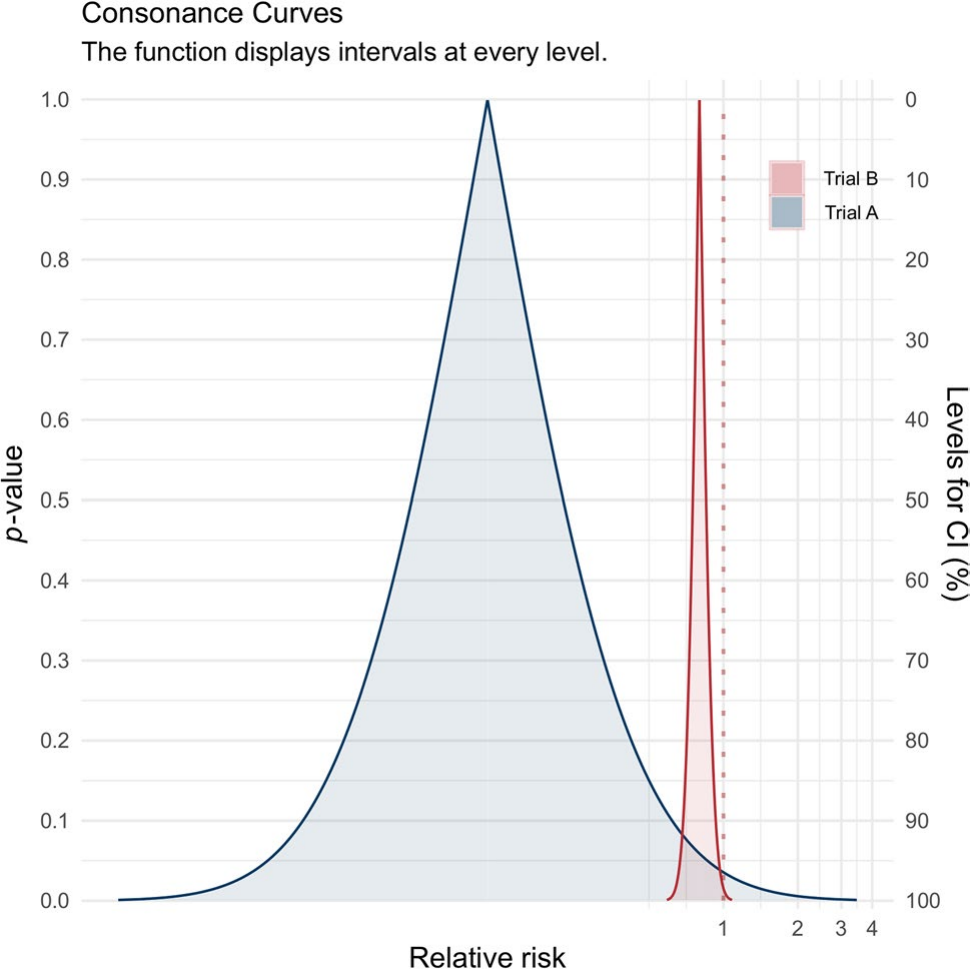
Consonance curves for both hypothetical trials

We may use the example by *Potter* [11] to clarify this: in a hypothetical trial A 1 of 100 patients experienced a negative event in the treatment group and 9 of 100 patients did so in the control group, which resulted in a relative risk of 0.11 in the treatment group compared to the control group with *P* = 0.02. In another hypothetical trial B, 200 of 4000 patients in the treatment group and 250 of 4000 patients in the control group experienced a negative event, resulting in a relative risk of 0.8, also with *P* = 0.02. The fragility index of the hypothetical trial A is 1, but the hypothetical trial B has a fragility index of 9. If we plot the consonance curves of the *P*-values of both studies using R’s [12] concurve-package (version 2.7.7) [13] (Fig. 1), it becomes obvious that hypothetical trial A is farther from the null effect of a relative risk of 1 compared to hypothetical trial B. Due to the much smaller sample size—reflected in the Fig. 1 by the wider confidence intervals—the very same *P* value of 0.02 in both studies can only occur due to a stronger effect. This relevant clinical difference between both trials is not captured by the fragility index, which considers the more relevant trial to be more “fragile” due to smaller number of events necessary to overturn the statistical significance.

We are aware that this is a rather technical argumentation, but as the recent controversy around posthoc-power [14] has shown, statistical details matter. There is an intrinsic fallacy regarding the concept of fragility in our specialty. Research in paediatric surgery is limited by the small numbers of major congenital malformations, the rarity of events [15]. Thus, paediatric surgery will always be penalised by the fragility index due to the diseases it deals with, which can be demonstrated by the example of severe isolated congenital diaphragmatic hernia: a recent randomised controlled trial for this highly effective treatment [16, 17] randomised 20 patients to foetal endoscopic tracheal occlusion and 21 patients to standard postnatal care. In the intention-to-treat-analysis, the survival rate at the age of six months with fetoscopic treatment was 50% (10/20), but 4.8% (1/21) in the control group (*P* = 0.0036) [18]. The trial would still be considered fragile, because it has a fragility index of 3, despite a massive clinical difference of a tenfold relative risk of death in the control group compared to foetal endoscopic tracheal occlusion.

We therefore thank *Saikia* & *Thakuria* for the opportunity to emphasise this highly relevant point of penalisation of small studies by the fragility index for paediatric surgeons with a relevant example. Therefore, our criticism of the fragility index was based on its statistical properties, whose inadequacies have been exposed by simulation studies before [11, 19, 20]. We therefore stand by our conclusion that the fragility index should be interpreted critically and may not be appropriate for paediatric surgery.
